# Palestinian doctors’ views on patient-centered care in hospitals

**DOI:** 10.1186/s12913-018-3573-0

**Published:** 2018-10-11

**Authors:** Wasim I M Sultan, Mutaz I M Sultan, José Crispim

**Affiliations:** 1 School of Administrative Sciences, PPU-Palestine & NIPE-Portugal, Hebron, Palestine; 2 School of Medicine, Al-Quds University, Al Quds, Palestine; 3 INESC TEC, Porto, Portugal

**Keywords:** Doctor-patient relationship, PCC, Doctors’ views, Palestinian hospitals

## Abstract

**Background:**

Understanding the perceived importance of Patient-Centered Care (PCC) among Palestinian doctors and how the provider and other clinical characteristics may impact their views on PCC is essential to determine the extent to which PCC can be implemented. This study investigates the provision of PCC among hospital doctors in a developing and unstable country, namely, Palestine.

**Methods:**

This descriptive, cross-sectional research employed self-report survey among 369 Palestinian doctors working in hospitals in 2016. Respondents completed the Provider-Patient Relationship Questionnaire (PPRQ) and were asked to rate the importance of 16 PCC subjects in a context-free manner. Then they scored the existence of eight contextual attributes in their workplace.

**Results:**

Although 71.4% of the participants got training in communication, only 45% of the participants knew about PCC. 48.8% of doctors considered the “exchange of information” with patients most important PCC component. Clustering identified three groups of doctors: 32.4% of doctors reported good perceptions of PCC, 47.5% moderate; and 20.1% poor. Older, married, and specialist doctors and those familiar with PCC are more likely classified in the “good” cluster. Results revealed a significant difference between doctors’ views based on their gender, experience, marital status, previous knowledge about PCC, and type of hospital in favor of males, experienced, married, familiar with PCC, and doctors in private hospital respectively. The level of job interest, nurses’ cooperation, the tendency of patients to hide information, and doctor’s friendly style were positively related with more perceived importance of PCC.

**Conclusion:**

We identified benchmark doctors who perceive the high relative importance of PCC. Our results highlighted knowledge gaps and training weaknesses among doctors in public and private hospitals in respect to their views on PCC. Decision makers may invest in the determined contextual predictors to enhance attitudes towards PCC. This work doesn’t address patients’ views on PCC.

**Electronic supplementary material:**

The online version of this article (10.1186/s12913-018-3573-0) contains supplementary material, which is available to authorized users.

## Background

There is ample evidence to support that implementing Patient-Centered Care (PCC) enhances health outcomes in different ways: improved patient satisfaction, enhanced behavior change, building trust, improving patient adherence, better providers’ clinical accuracy, and more active patient self-management [1–13]. From an economic perspective, gains through fewer diagnostic tests, fewer referrals, and less cost of nonadherence also can be achieved when implementing PCC [14, 15].

PCC is well documented as a key attribute of high-quality healthcare services [10, 16]. The Institute of Medicine considered the PCC as one of the six essentials of the quality of care in the twenty-first century [8, 17]. The PCC approach emphasizes the effective provider-patient relationship and addresses better understanding of patient’s own feelings, preferences, and views about the disease and treatment [18]. The fundamental characteristics of PCC, as identified in the literature, are the patient involvement in care and the individualization of patient care [14].

From a clinical practice perspective (applied in this research project), PCC refers to providing a provider-patient relationship that integrates the well-being of the individual and the psychosocial context of patients [19]. The psychosocial aspects of care (the patient’s values, emotions, needs, expectations, and preferences) are considered to guide clinical decisions [17]. A common understanding of the patient’s condition will help the provider getting the relevant information and execute the best possible clinical strategy [11]. Therefore, patients are more likely to receive the maximum achievable benefits [20, 21].

To date, no studies have examined the Palestinian doctors’ views on the provision of PCC, therefore, our work addresses a major gap in the literature by empirically investigating the perceived importance of PCC among doctors in a developing and unstable country, namely, Palestine. Previous studies are limited to describe the health reform initiatives in Palestine [22, 23] or to describe the status of healthcare [24–30]. To elaborate further on the challenges of implementing PCC, we examine the associations between doctors’ views and contextual factors (structural attributes in the workplace). Therefore, our work has the potential to determine the extent to which PCC can be implemented in the Palestinian context and help to capture the areas that merit collaborative institutional efforts for enhancing the PCC knowledge and practice.

To sum up, because PCC is an essential attribute of the high-quality healthcare system, and because the provider-patient relationship is an area of significant concerns in the East Mediterranean Region [31], our intention is to address two major research objectives:

1. Assess the perceived importance of implementing PCC among the Palestinian doctors in hospitals.
2. Explore provider and other clinical characteristics that may have an association with doctors’ views on PCC.

### The case of Palestine

Behind the daily life of the Palestinians lies an exceptional set of factors that drives their behaviors and contributes to their performance. Palestinians are stressed by an environment characterized by chronic conflict with the Israelis, worsening economic conditions, restrictions on movement, and the gap between the reality of occupation and the yet to be the State of Palestine [32–34]. While acting in a stressful environment, Palestinian hospital doctors are challenged to cope with professional practices. Therefore, understanding their views on the importance of delivering PCC serves the needs to determine the extent to which PCC can be implemented.

The Palestinian health care system is politically framed to serve previous commitments and liberation promises of the public authorities rather than being socioeconomically structured [35]. For example, although public hospitals suffer from understaffing, shortages of supplies and high rates of bed occupancy [27], the applied health insurance schemes cover most of the population and increase the burden on the limited public services [29]. Therefore, it is believed that the emphasis on quantity preceded the quality of care in the Palestinian hospitals [26].

Perhaps gauging the growing problems in the public hospitals, the Palestinian Ministry of Health (MoH) allocates more than 35% of its budget to purchase hospital services from the private hospitals [36]. While the decision seems to have had a favorable impact on better access, quality is compromised. The MoH is loaded with receivables they can’t reimburse for the private hospitals, as a result, the private hospitals suffer financial deficit to meet salary payments on a regular basis and their employees (including doctors) are less induced to do any more than what is minimally required. This complex context may affect doctors’ views on PCC [16, 37]. Hence, exploring what are the provider and other clinical characteristics that may contribute to doctors’ views on PCC is also an important topic?

### The scope of the study

PCC is a multidimensional concept, regrettably, the literature lacks consensus on a standard list of PCC components [38], but, previous studies tend to focus on components of PCC like the patient’s agenda [39], information, communication, and education [7, 17], or shared decision making [40].

The scope of our work is examining the perceived importance of sixteen PCC actions during the medical interview among doctors in Palestinian hospitals. These actions represent four PCC components. Then, to examine the association between the perceived importance of these components and eight contextual factors. The PCC components are: (1) Exchange of information such as listening to the patient, asking questions, giving clear information and using a quiet tone; (2) Individualization such as showing interests in the patient’s feelings, wants, expectations, and knowledge; (3) Empathy which represent the doctor’s ability to respond to and improve his patient’s emotional state [6], evidence shows that doctor’s empathy and clinical outcomes are positively related [41]; and (4) Patient involvement such as engaging the patient in informed and shared decision making. To achieve this research objective, we applied the Provider-Patient Relationship Questionnaire (PPRQ) which was developed and tested by Gremigni et al. (2016); a survey that contains 16 scale self-report items [18]. Two recent works have also taken this approach to investigate PCC among healthcare professionals in Italian hospitals [18, 42].

To identify contextual factors that may have effects on the doctors’ views on PCC, we reviewed the relevant literature [7, 43–45]. Guided by the literature and building on Epstein and Street work, “*Patient-Centered Care is a quality of personal, professional, and organizational relationships …”* [46] we discussed with two doctors an initial list of eleven contextual factors. Then, we conducted a pilot survey of ten doctors who confirmed only eight contextual factors and the relevance of PPRQ. These eight contextual subjects capture three sets of potential factors: (1) Organizational factors (structural attributes in the workplace) that include administrative support, workload conditions, and nurses’ cooperation [47]. (2) Attributes related to the doctor in the workplace such as job satisfaction, job interest, and his tendency to approach patient’s problem either formally or friendly [7, 20, 48–50]. (3) Attributes related to the patient include the level of health literacy and tendency to hide relevant information [51–53]. The doctors are asked to score the prevalence of eight contextual factors.

## Methods

We conducted secondary research to find studies on PCC, health education, communication, or doctor-patient relationship in Palestine. We found few published studies, none of them addressed the provision of PCC. Our research project will answer the following research questions:

- RQ1: What is the relative importance of 16 PCC actions (four PCC components) as perceived by the Palestinian doctors?
- RQ2: What are the PCC actions or doctor’s background that significantly classify Palestinian doctors into clusters (like-minded doctors) based on their views on the importance of PCC?
- RQ3: Do Palestinian doctors perceive differently the importance of PCC components based on their socio-demographic characteristics or based on the type of hospital they work for?
- RQ4: What are the contextual factors related to the perceived importance of PCC components among Palestinian doctors?

### Research design

This descriptive, cross-sectional research used self-report survey among Palestinian doctors working in hospitals. The assessment of attitudes often uses questionnaires and self-report data [54]. Self-report has been identified as a key aspect of medical professionalism [55]. It provides insights into the doctor’s thoughts, feelings, and emotions towards the patient-provider relationship [7]. This method is common in the literature to explore doctors’ views and professionals in the workplace domains [56–59].

### Questionnaire design

The questionnaire is divided into four sections (see Additional file 1): the socio-demographic characteristics of participants; measures of 16 PCC actions (subjects); measures of eight contextual factors (structural attributes); and four measures of the Socially Desirable Response Set (SDRS) to test for participants’ propensity to give socially desirable answers (adopted from the literature) [60].

The first section includes the sociodemographic characteristics of the participants (Table 1). It is assumed that doctors approach their patients’ problems in different ways and according to the doctor’s experience levels, knowledge, background, and beliefs. Therefore, this part describes the sample characteristics and helps to explore differences among doctors based on their characteristics with respect to their views on the relative importance of PCC components.

**Table 1:** Sample characteristics (*N* = 369)

Characteristic	Counts (%)
Profession	
Specialist doctor	139 (37.7)
Resident doctor	138 (37.4)
General doctor	92 (24.9)
Hospital Department	
Surgery	71 (19.2)
Internal Medicine	52 (14.1)
Obstetrics & Gynecology	34 (9.20)
Pediatrics	69 (18.7)
Anesthesia	08 (2.20)
Emergency	07 (1.90)
Radiology	14 (3.80)
Other departments	114 (30.9)
Gender	
Male	276 (74.8)
Female	93 (25.2)
Marital Status	
Unmarried	143 (38.8)
Married	226 (61.20
Age	
≤ 35 years	222 (60.20
> 35 years	147 (39.2)
Experience	
≤ 5 years	218 (59.1)
> 5 years	150 (40.7)
University of Bachelor	
Inside Palestine.	137 (37.1)
Outside Palestine.	229 (62.9)
Communication Training	
Never got training	102 (27.6)
Got training	262 (71.4)
Knowledge about PCC	
Don’t know about PCC	203 (55)
Knows about PCC	162 (45)
Hospital settings	
Public	181 (49.1)
Private	188 (50.9)
Total	369 (100)

The second section employs the Provider-Patient Relationship Questionnaire (PPRQ) and includes sixteen subjects of interest that represent four PCC components [18]. We used a five-point Likert scale to score the level of perceived importance and ranging from “not at all important” to “totally important”, responses were coded from “1” to “5” respectively (Table 2). The doctors are asked to report the importance of these actions in a context-free manner.

**Table 2:** Descriptive statistics of PCC items and components

Constructs and survey items (Cronbach’s α = 0.911)	N	M	SD
Exchange of information (Cronbach’s α = 0.789)	365	4.00	0.76
Provide clear information to the patient.	369	3.95	1.01
Turn to the patient in a calm and quiet tone	369	3.86	0.95
Show respect to the patient as a person rather than a case.	368	4.15	0.98
Pay attention to what the patient says.	366	4.04	0.94
Individualization (Cronbach’s α = 0.816)	357	3.60	0.78
Show interest in what the patient feels about his status.	366	3.81	0.92
Show interest in what the patient knows about his disease/prognosis.	365	3.62	1.02
Show interest in what the patient wants from care.	366	3.58	0.98
Show interest in what the patient expects from care in this hospital.	365	3.42	0.97
Empathy (Cronbach’s α = 0.747)	360	3.59	0.77
Understand the emotions that the patient may have.	366	3.68	0.94
Check how illness affects the patient’s daily life activities.	368	3.70	0.97
The doctor acts as putting himself in the “patient’s place”.	366	3.24	1.12
Inspire confidence and security when touching the patient or being nearby.	365	3.75	1.04
Patient involvement (Cronbach’s α = 0.796)	364	3.61	0.78
Give the patient enough time to ask and talk about his disease.	368	3.67	0.96
Ask the patient questions that allow him to express his point of view.	367	3.53	0.98
Give him encouragement and transmit optimism.	368	3.85	0.96
Offer the patient an opportunity to discuss and share decision making.	368	3.40	1.05

1 = not at all important (minimum score), 5 = mostly important (maximum score)

The third section includes eight factors related to the working place domain (less controlled by the doctor) and related to hospital administration, other professionals (nurses), and the patients. In addition to job interest, job satisfaction, and level of doctor’s formal style to communicate with patients.

We used this preliminary version and conducted a pilot study of ten participants. One PCC item was modified to suit the Palestinian social context, and three irrelevant contextual factors were eliminated, in the result, eight contextual factors were analyzed.

The patient-related factors were discussed and confirmed as they may influence the medical interview process [51] for two reasons: First, because it is not uncommon in developing countries that patients hide information about their disease due to perceptions of having social harm in the future; Second, it is assumed that low levels of health literacy in developing countries’ setting can impede understanding health information.

### Participants

In 2016, we collected data from doctors working in public and private hospitals in the West Bank and East Jerusalem. We followed a convenient sampling technique. The English printed version of the self-administered questionnaire (Additional file 1) targeted all the full-time hospital employed doctors in the West Bank and East Jerusalem (*N* = 1418). Doctors in psychiatric hospitals were excluded. We obtained ethical approval from the Palestinian Ministry of Health to carry out the research. We started to recruit participants to fill out the questionnaire online, but we failed. Then, using a printed English version of the questionnaire, we asked doctors to participate on a voluntary basis during the 3 days of the National Medical Conference. The conference was organized by the Palestinian Medical Association at the City of Bethlehem in West Bank in October 2016. Doctors were asked to remember recent few medical interviews and how they interacted with their patients, a brief introduction about the research was also explained to each participant. We distributed 590 surveys, 373 were returned (63%). We excluded four disqualified surveys (either incomplete or used socially desirable answers) then we analyzed the data of (369) respondents.

### Data analysis techniques

Several data analysis techniques were used to answer the proposed research questions. Data were analyzed at a 95% confidence interval (α = 0.05) using the SPSS 23. Descriptive statistics (RQ1), multivariate analyses of MANOVA (RQ3) and canonical correlation techniques (RQ4). In addition to cluster analysis and Chi-square analysis (RQ2). Each PCC component was assessed for reliability using Cronbach’s Alpha. The coefficients of the “exchange of information”, “individualization”, “empathy”, and “patient involvement” were α = 0.79, 0.82, 0.75, and 0.8 respectively [61].

## Results

Table 1 summarizes the demographic characteristics of the participants. About 50% of the Participants were from public hospitals. Private hospitals could be owned by the not-for-profit organization or owned by the for-profit organization but not owned by the government. About 63% of the participants got their education outside the country. Surprisingly, although 71.4% got training in communication, only 45% of the participants knew about PCC.

Most of the respondents were male doctors (74.8%). It is worth noting that the youth two medical schools in West Bank created a better chance for females to become doctors without traveling abroad and to benefit from joining the profession of medicine. Even though, the gender gap is still common in the Palestinian hospitals (25% of the participants are females).

### The reported perceived importance of PCC components (RQ1)

On average, 48.8% considered the exchange of information with patients as “most important” or “totally important” and only 27% of doctors considered the other three PCC components as most or totally important. Previous evidence reported low levels of implementing PCC; The Agency for Healthcare Research and Quality (AHRQ) reported that only around 45% to 62% of the patient encounters “always” used the PCC approach in developed countries [45, 62]. Palestinian doctors reported less frequent perceived importance than the previously published levels of implementing PCC. Results are shown in Table 2.

On average doctors reported high importance of “exchange of information” component (*M* = 4) than “patient involvement” components (*M* = 3.64), then “individualization” component (*M* = 3.6), and finally, the “empathy” component scored the lowest perceived importance (*M* = 3.59).

### Doctors differ in their views with respect to seven PCC subjects (RQ2)

**Fig. 1:**
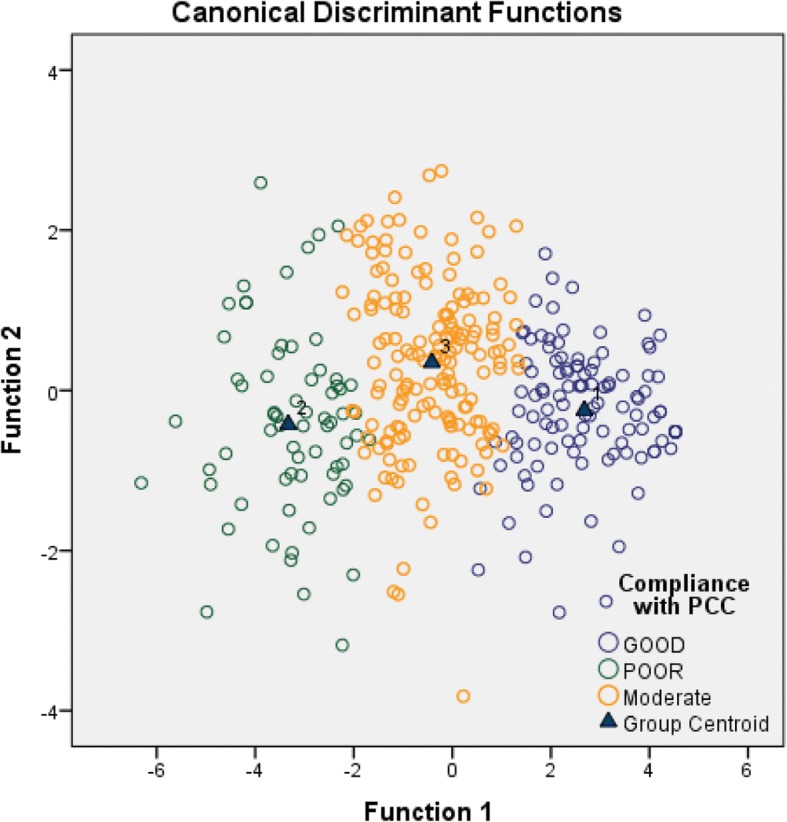
Three groups of doctors based on their views on PCC

We conducted cluster analysis to find the underlying structure of relationships among the doctors based on their views about the PCC. The two-step approach for clustering (hierarchical then K-mean cluster analyses) formed three groups of doctors (Fig. 1). Further, a discriminant analysis of the outcomes was conducted, two canonical discriminant functions predicted the cluster outcomes, the model significantly discriminated the clusters, Wilks’ Lambda = 0.158, *p* <  0.01. Function 1 explained 97.6% of the variance between clusters, the canonical correlation coefficient was 0.908. The standardized canonical discriminant function 1 coefficients assigned high weights (> 0.2) to seven PCC subjects which were significant in differentiating the clusters (Table 3). Improving the perceived importance of these seven subjects could be the focus of investment in training.

**Table 3:** Cluster centers: seven subjects differentiate doctors based on their views on PCC

PCC items	PCC components	High (32.4%)	Moderate (47.6%)	Poor (20%)
1. Provide clear information to the patient	Exchange of information	5	4	3
2. Show respect to the patient as a person rather than a case		5	4	3
3. Show interest in what the patient knows about his disease.		4	4	3
4. Show interest in what the patient expects from this hospital	Individualization	4	3	3
5. Understand the emotions that the patient may have	Empathy	5	4	3
6. Give the patient enough time to ask and talk about his/her disease	Patient involvement	4	4	3
7. Offer the patient an opportunity to discuss and share decision		4	3	3

Respondents’ characteristics were cross-tabulated by the three clusters (2 × 3 model). The data was analyzed using a *chi-square* test of independence to look for an overall significance of the relationship between the doctor’s background and the PCC cluster in which she was classified (Table 4). We used the value of standardized residuals (the difference between observed and expected value is (> 1.196 or < − 1.196)) to find which demographic attribute has the most contribution to the significance of the relationship.

**Table 4:** Descriptive associations between respondents’ demographic characteristics and PCC behavioral clusters

		N	PCC Behavioral Clusters			X^2^	Sig.^a^	Most Contribution Standardized Residual
			GOOD %	POOR %	Moderate %		p	
Job title	Specialist	129	41.9	18.6	39.5	9.53	**0.049**	**Specialist X Good = 1.9**
	Resident	127	26.8	25.5	60.4			
	GP	087	26.4	18.4	55.2			
Gender	Male	258	33.7	17.4	48.8	4.67	0.097	**Female X Poor = 1.7**
	Female	085	28.2	28.2	43.5			
Age	≤ 35 years	204	26.5	21.6	52.0	8.01	**0.018**	**> 35 years X Good = 1.8**
	> 35 years	139	41.0	18.0	41.0			
Marital Status	Single	133	24.1	21.8	54.1	6.39	**0.031**	**Single X Moderate = 1.1**
	Married	210	37.6	19.0	43.3			**Married X Good = 1.3**
Hospital	Public	165	24.8	23.6	51.5	8.57	**0.014**	**Residuals are < 1.1**
	Private	178	39.3	16.9	43.8			
Experience	≤ 5 years	200	28.5	20.5	51.5	3.54	0.17	**> 5 years X Good = 1.2**
	> 5 years	142	38.0	19.0	43.0			
Bachelor	Local	129	30.2	20.9	48.8	0.59	0.74	
	Abroad	211	34.1	19.0	46.9			
Training	Untrained	094	30.9	26.6	42.6	3.92	0.14	**Untrained X Poor = 1.5**
	Trained	245	33.1	17.1	49.8			
Familiar with PCC	Unfamiliar	185	28.1	25.4	46.5	8.02	**0.018**	**Unfamiliar X Poor = 1.6**
	Familiar	154	37.0	13.6	49.4			

^a^Significant relationships are indicated in boldface

Job title, age, marital status, hospital type, and familiarity with PCC were significantly associated to which cluster the doctor was located. The specialists, the aged, the married, the doctors in private hospitals, and the familiar doctors with PCC were more frequently classified by the cluster of the more perceived importance of PCC. The lack of familiarity with PCC and lack of previous training contributed most to poor views about PCC among doctors who were neither familiar with PCC nor trained. However, gender, experience, university of bachelor, or previous training had no significant effect (*p* > 0.05) on which PCC cluster the doctor was classified.

### Palestinian doctors perceive differently the importance of PCC components based on their socio-demographic characteristics or the type of hospital they work for (RQ3)

Multivariate techniques were adapted because they allow for simultaneous comparisons among the variable sets and limit the probability of committing type I error in the study [63]. The approach also provides congruence between the explanatory nature of the study and the consideration of multiple possible causes on multiple effects. The scores of each set of four items that represent a certain PCC component were averaged and became a mean index for that PCC component. The new four PCC scores represent the combined dependent variables (DVs) of the study through the subsequent carried out statistical analyses. Descriptive statistics appear in Table 5. Pearson correlation between the four variables was substantial for all pairs. Empathy was the most strongly correlated with patient involvement and exchange of information (*r* = 0.67), while patient involvement was the least correlated process (*r* = 0.5) with the exchange of information.

**Table 5:** Descriptive statistics of four PCC components (mean indexes)

PCC Construct	N	Min.	Max.	Mean	SD
- Exchange of Information	365	1.00	5.00	4.00	0.76
-Individualization	357	1.25	5.00	3.62	0.78
-Empathy	360	1.25	5.00	3.59	0.77
-Patient Involvement	364	1.25	5.00	3.61	0.78

We carried out MANOVA tests to detect whether the combined dependent variables (DVs) differ across groups of participants based on their demographic attributes (Table 6). Post hoc tests detected differences between subjects and tested for the effects of each group of respondents on each PCC component (DV). Box’s M test indicated that the assumption of equality of covariance matrices was met *p* > *0.05*. Levene’s test showed that the assumption of equality of error variance was met *p* > 0.05, equality of error variance of DVs across groups was supported.

**Table 6:** The associations between the categorical variable (socio-demographic) and the PCC components (MANOVA tests)

Groups		Wilk’s Λ	Test	Partial η^2^	In favor of
Specialists, residents, GPs	Combined DVs	0.964	F(8,674) = 1.58, p = 0.127	0.018	–
	-Exchange of Information		F(2,343) = 1.99, p = 0.139	0.012	–
	- Individualization		F(2,343) = 4.630, **p = 0.01**^**a**^	0.027	Specialists
	- Empathy		F(2,343) = 1.930, p = 0.14	0.011	–
	- Involvement		F(2,343) = 2.231, p = 0.10	0.013	–
Male, female doctors	Combined DVs	0.959	F(4,338) = 3.65, **p = 0.006**	0.041	males
	-Exchange of Information		F(1,343) = 0.30, p = 0.578	0.001	–
	- Individualization		F(1,343) = 4.97, **p = 0.026**	0.014	Males
	- Empathy		F(1,343) = 0.080, p = 0.77	0.000	–
	- Involvement		F(1,343) = 7.28, **p = 0.007**	0.021	Males
Married, unmarried doctors	Combined DVs	0.971	F(4,338) = 2.50, **p = 0.042**	0.029	Married
	-Exchange of Information		F(1,343) = 0.80, p = 0.372	0.002	–
	- Individualization		F(1,343) = 5.70, **p = 0.017**	0.017	Married
	- Empathy		F(1,343) = 5.48, **p = 0.020**	0.016	Married
	- Involvement		F(1,343) = 7.89, **p = 0.005**	0.023	Married
Experience > 5, ≤ 5 years	Combined DVs	0.965	F(4,337) = 3.08, **p = 0.016**	0.035	Experienced
	-Exchange of Information		F(1,341) = 0.20, p = 0.653	0.001	–
	- Individualization		F(1,341) = 7.90, **p = 0.005**	0.023	Experienced
	- Empathy		F(1,341) = 1.68, p = 0.195	0.005	–
	- Involvement		F(1,341) = 6.01, **p = 0.015**	0.017	Experienced
Local education, abroad	Combined DVs	0.989	F(4,335) = 0.950, p = 0.43	0.011	–
	-Exchange of Information		F(1,338) = 0.34, p = 0.558	0.001	–
	- Individualization		F(1,341) = 0.67, p = 0.414	0.002	–
	- Empathy		F(1,341) = 0.07, p = 0.796	0.000	–
	- Involvement		F(1,341) = 2.50, p = 0.112	0.007	–
Trained, untrained doctors	Combined DVs	0.979	F(4,334) = 1.80, p = 0.127	0.021	–
	-Exchange of Information		F(1,337) = 0.16, p = 0.694	0.000	–
	- Individualization		F(1,337) = 1.75, p = 0.178	0.005	–
	- Empathy		F(1,337) = 5.25, **p = 0.023**	0.015	Trained
	- Involvement		F(1,337) = 4.33, **p = 0.038**	0.013	Trained
Familiarity with (PCC)	Combined DVs	0.968	F(4,334) = 2.75, **p = 0.028**	0.032	Familiar
	-Exchange of Information		F(1,337) = 2.39, p = 0.125	0.007	–
	- Individualization		F(1,337) = 7.27, **p = 0.007**	0.021	Familiar
	- Empathy		F(1,337) = 4.67, **p = 0.031**	0.014	Familiar
	- Involvement		F(1,337) = 9.98, **p = 0.002**	0.029	Familiar
Public, private hospital	Combined DVs	0.968	F(4,338) = 2.75, **p = 0.028**	0.037	Private
	-Exchange of Information		F(1,343) = 11.8, **p = 0.034**	0.034	Private
	- Individualization		F(1,343) = 6.09, **p = 0.014**	0.018	Private
	- Empathy		F(1,343) = 3.49, p = 0.063	0.010	–
	- Involvement		F(1,343) = 6.18, **p = 0.013**	0.018	Private

^a^Significant effects are indicated in boldface

Results show that the combined dependent variables (DV) are not significantly different, in the general sense, across the three job titles (specialists, residents, and general doctors, *P* > 0.05). However, the main effect of job title on the “individualization” component was significant (Wilk’s Lambda = 0.964, F (2,343) = 4.630, *p* = 0.01), the effect size of job title was 0.027. Scheffe’s multiple comparisons indicate that there is a significant difference in perceived importance of “individualization” (*p* = 0.018) between specialists and residents in favor of specialists.

Male doctors reported the more perceived importance of PCC components than female doctors. The combined DVs were significantly different across gender groups, the effect size was 0.041. The main effect of gender on the “individualization” component was significant (*p* = 0.026). The main effect of gender on “patient involvement” component was significant (*p* = 0.007).

Married doctors reported the more perceived importance of PCC components than unmarried doctors. The combined DVs were significantly different between married and unmarried doctors (*p* = 0.042). The main effect of marital status on “individualization”, “empathy”, and “patient involvement” components was significant.

Experienced doctors (with more than 5 years of experience in a hospital) reported the more perceived importance of PCC components than other doctors. The combined DVs were significantly different across the two groups. The main effect of experience on “individualization” component was significant (*p* = 0.005). The main effect of experience on “patient involvement” component was significant (*p* = 0.017).

Results show no significant differences between doctors’ views on PCC components based on the place of education whether they studied in the Palestinian Medical Schools or abroad. Likewise, results show that previous training in communication skills had no significant effect, in the general sense, on the combined DVs (*P* > 0.05). However, the main effect of training on “empathy” component was significant (*p* = 0.023). The main effect of training on “patient involvement” component was also significant (*p* = 0.038).

Familiar doctors with PCC (having previous knowledge about PCC) reported more importance of PCC components than doctors who were not familiar with PCC. The combined DVs were significantly different across the two groups (*p* = 0.028). The main effect of familiarity with PCC on “individualization” component was significant (*p* = 0.007). The main effect of knowing PCC on “empathy” component was significant (*p* = 0.031). The main effect of experience on “patient involvement” component was significant (*p* = 0.002).

Finally*,* doctors in private hospitals reported the more perceived importance of PCC components than doctors in the public hospitals (Table 6). The combined DVs were significantly different across the two groups (Wilk’s Lambda = 0.968, F (4,338) = 2.75, p = 0.02). The effect size was 0.037. The main effect of hospital setting on the “exchange of information” component was significant (F (1,343) = 11.8, *p* = 0.034). The effect size was 0.034. The main effect of hospital setting on “individualization” component was significant (F (1,343) = 6.09, *p* = 0.014), the effect size was 0.018. The main effect of hospital types on “patient involvement” component was significant (F (1,343) = 6.18, *p* = 0.013), the effect size was 0.018. But there was no significant effect of hospital type on “empathy” component (*p* = 0.063).

### The relation between the presence of contextual factors and doctors’ views on PCC (RQ4)

The projected eight contextual attributes were measured on a five-point Likert scale ranging from “totally disagree” to “totally agree” and coded from one to five respectively (Table 7).

**Table 7:** Scores and descriptive statistics of the proposed eight contextual factors

Contextual barrier^a^	N	Mean	SD
Hospital culture does not support effective medical interview.	367	3.07	1.10
I am overloaded, and I don’t have enough time for a good interview.	368	3.24	1.11
Nurses cooperate positively.	364	2.78	0.99
I feel satisfied with my work in this hospital (N).	362	2.69	0.99
I find my job as interesting (N).	367	2.11	0.93
I prefer to be formal rather than warm and friendly.	369	2.83	1.14
Patients tend to give less information due to social consequences.	366	3.48	0.94
Most patients are not aware of their health status or their disease.	365	3.44	1.07

^a^(1) = strongly disagree, (5) = strongly agree. N, negatively worded item

We conducted a canonical correlation analysis (CCA) using the set of eight contextual factors as a predictor variable set of the four PCC components as the criterion variable set (given in Table 5). The purpose was to answer the study question whether doctors’ working conditions are predictive of the perceived importance of PCC components by evaluating the multivariate relationship between the two variable sets. The four PCC components of interest are the dependent variable set (DVs).

The analysis yielded four functions with squared canonical correlations (Rc^2^) of .213, .047, .011 and .009 respectively. Collectively, the full CCA model (*N* = 331) was statistically significant, with a Wilk’s λ of (0.73), F (32, 1178) = 3.21, *p* <  0.001 and explained (1- λ = 27%) of shared variance between the two variable sets. Thus, the r^2^ type effect size was 0.27 [63]. The dimension reduction analysis allowed for hierarchal tests for significance and revealed that the full CCA model (Functions 1 to 4) was statistically significant F (32, 1178) = 3.2, *p* <  0.001. Functions 2 to 4, 3 to 4, and function 4 tests did not explain a statistically significant amount (*p* > 0.05) of shared variance between the variable sets. Therefore, we considered the effect of function 1 to identify the contributing variable as it captured most of the shared variance and not to interpret the effect of other functions as they only explained less than 5% of the variance.

Table 8 presents the standardized canonical function coefficients and structure coefficients for Function 1. Structure coefficients are used to decide what variables are contributing to this relationship. Looking at the coefficients, all the PCC components have a contribution as supported by the large squared structure coefficients (r_s_^2^), which represents the shared variance between the observed PCC scores and the variate created from the observed variable’s set.

**Table 8:** The standardized canonical function coefficients

Criterion Variables (DVs)		Function 1	
	Coef.	r_s_	r_s_^2^ (%)
Exchange of information	- 0.454	- 0.851	72.4
Individualization	- 0.107	- 0.778	60.5
Empathy	- 0.215	- 0.819	67.1
Shared decision	- 0.413	- 0.855	78.3
Predictor Variables (IVs)			
Administration support.	- 0.040	+ 0.182	3.30
Workload.	+ 0.040	+ 0.172	3.00
Nurses’ cooperation.	- 0.349	- 0.475	22.5
Level of job satisfaction	+ 0.087	- 0.358	12.8
Level of job Interest.	- 0.729	- 0.696	48.4
Doctor’s formal style.	+ 0.574	+ 0.530	28.1
Patients tend hide information.	- 0.266	- 0.169	2.90
Low level of health literacy.	+ 0.148	+ 0.050	0.25

However, the four predictor variables job interest, doctor’s style, nurses’ cooperation, and the patient’s tendency to hide information were the primary contributors to the predictor variate (Table 9). The doctor’s style predictor was negatively related to all the PCC components which support the theoretically expected relation, the more formal the doctor interviews his patients, the less he considers PCC as important. The other three predictors were positively related.

**Table 9:** Regression analysis and causality relationships

Significant Predictor Variables	Exchange of Information		Individualization		Empathy		Involvement	
	β	*p*	β	*p*	β	*p*	β	*p*
Level of job interest	0.34	< 0.001	0.24	< 0.001	0.21	< 0.001	0.27	< 0.001
Doctor’s formal style	- 0.23	< 0.001	- 0.25	< 0.001	- 0.22	< 0.001	- 0.21	< 0.001
Nurses’ cooperation	0.06	0.252	0.13	0.02	0.18	0.001	0.19	0.001
Patient’s tendency to hide information	0.12	0.026	0.05	0.33	0.11	0.047	0.10	0.080

The results of the regression bivariate analysis supported the analysis of standardized canonical function coefficients. Beta weights reflected the relative contribution of each predictor to the PCC processes [63]. The relationship was significant to all the four predictors (with high canonical function coefficients) of the four PCC components. Two exceptions involve no significance, the Patient’s tendency to hide information with “individualization” component and the nurses’ cooperation with the “exchange of information” component.

PCC behaviors were significantly associated with the adopted personal style of the doctors. Doctors who adopted a formal style rather than a warm, friendly style were negatively correlated with PCC components. The four PCC components were significantly negatively correlated (*p* <  0.001). Pearson coefficients were - 0.2, − 0.24, − 0.20, − 0.19 respectively.

## Discussion

The first research objective of this study was to assess the doctors’ perceived importance of PCC among hospital doctors. The remarkable finding was the prevalence of unfamiliarity with PCC (55% of the Palestinian doctors never knew about PCC). These results were observed by other researchers in developing countries [50], where it is not uncommon that the patients’ voice is ignored by care provider [64].

The perceived importance of PCC among Palestinian doctors was significantly different between doctors in private and doctors in public hospitals in favor of doctors in private hospitals. Doctors in public hospitals were more frequently classified in poor and moderate clusters of doctors based on their perceived importance (23.6% and 51.5% respectively). MANOVA tests supported the same result. Previous evidence also supported our results as the state of public hospitals in Palestine overcrowded and suffer understaffing. Professionals work in crisis-mode and perceive medical interview as time-consuming [26, 27]. Others found that, in developing countries, private hospitals are competing and induced to provide better services than public ones [65].

Doctors, based on their views on PCC, were classified into three groups (1) the good cluster, highly recognize the importance of PCC (2) the poor cluster, and (3) the moderate cluster. Doctors were significantly differentiated into clusters by seven PCC subjects, investment decisions in training should stress these seven PCC subjects. Meanwhile, a remarkable result indicated that the lack of training or lack of familiarity with PCC was statistically associated with the poor perception of the importance of PCC. Previous evidence showed that nearly all educational and training interventions had improved interpersonal skills among doctors [7]. Results show that the specialists, the aged, and the married doctors are more frequently in the cluster of good views. This study didn’t investigate the motives behind good views on PCC among these doctors, it may be hypothesized that good performers devoted self-developed social skills and individual professionalism to meet the patients’ psychosocial expectations.

In the general sense, the socio-demographic factors: significant differences exist across the groups of gender, marital status, experience, and familiarity with PCC in favor of males, married, experienced, and familiar with PCC doctors respectively (Table 6). Male doctors have the advantage of the more perceived importance of PCC than female doctors. This result is explained by the common culture among Palestinians and the gender gap for closer communication, further research could be useful.

Training in communication, in the overall sense, was not significantly associated with the more perceived importance of PCC. Yet, training is significantly associated with “empathy” and “patient involvement” components. Training should assist doctors to become more informative, empathic and mindful of moving their role as characterized by the authority to one that has the goals of the partnership, solidarity, and collaboration [46]. Our results are consistent with previous evidence which show significant differences between doctors based on personal differences or patient differences [49, 66–68].

Doctors who adopted formal style during the medical interview were less likely to exchange information with patients than doctors approaching their patients in a warm and friendly style. Further, a doctor’s formal style was negatively correlated to all PCC components. Literature suggests a consistent finding is that doctors with a warm and friendly style to be more effective in emotional and cognitive care [48].

The preliminary model of canonical correlation analysis discussed in this study provides insights to both hospital managers and policy makers who might use the results to improve implementing PCC. Improving PCC performance will enhance service quality in the hospital environment in Palestine. The model identified four significant predictable factors as perceived by the doctors that have significant effects on the four PCC processes of interest (Table 9). This is important because doctors’ perceptions have behavioral implications on their performance. Investments in managing these factors will have positive influences on patients as they will receive better care of the often-neglected psychosocial dimensions of hospital services.

Since all the four predictors had a significant association with the perceived importance of PCC components, managers might base PCC enhancement strategies based on the parameters of the model. The standardized βs provide straightforward answers to the targeted improvements. Addressing the job interest issue (β = 0.34 SD) will deliver the greatest improvement in the perceived importance of the “exchange of information” component. To formulate a strategy of four items to improve the perceived importance of “empathy” component by 1SD, it requires an increase of 0.21SD of job interest, an increase of 0.18SD of nurse collaboration, and an increase of perception among doctors that patients may hide relevant information due to social consequences of 0.11SD. In addition to decreasing the level of formality in doctor’s style by 0.18SD.

Findings showed patient-related factors that influence the doctor’s views. The perception on the doctor’s side about his patients can’t be ignored [53]. Surprisingly, the perception of low levels of health literacy had no significant influence on the doctor’s PCC behaviors. Additional research is needed to investigate health literacy in Palestine [52]. Second, the patients’ tendency to hide relevant information due to felt social consequences had promoted doctors to more PCC intention, increased perceived importance of “exchange of information” (β = 0.12), increased “empathy” (β = 0.11), and increased “patient involvement” (β = 0.10) Doctors who perceived this tendency reacted positively to improve patient engagement.

## Conclusion

While attention to improving the quality of services provided in Palestinian hospitals is growing, the problems of the health care delivery system are reported with increasing frequency in the public media. This work is the first research to investigate the consultation between doctor and patient among Palestinian doctors, little is known about the topic. The study identified factors that contribute to the doctors’ performance and highlighted the challenges of implementing PCC in a developing and unstable context, namely, Palestine.

While previous literature focused on how to implement PCC, this study investigated the vital components of the healthcare system that underpin effective PCC behaviors. This study introduced a prior knowledge about personal, professional and organizational aspects and their relationships with better views on PCC among Palestinian doctors in hospitals. It is of significance as it comes at a time when the Palestinian Ministry of Health is adopting a family practice model. Doctors are requested to have better attitudes towards PCC, this will help the public authorities to effectively adopt the family practice model as a strategy to manage the overcrowded state of work of the Palestinian hospitals.

Results highlighted gaps between public and private hospitals, managers of public hospitals can benchmark the private hospitals to enhance attitudes towards PCC. Results also suggest that both types of hospitals have room for improving their doctors’ interpersonal skills. Hospital managers might base PCC enhancement strategies on the parameters of the canonical correlation model and pay more attention for managing the identified predictors of PCC behaviors. The study is limited to doctors’ views on PCC and generates the question of patients’ perceptions and views to identify their informative and PCC needs within the Palestinian context.

## Additional file

Additional file 1: Palestinian Doctors’ Views on Patient-Centered Care in Hospitals. (DOCX 24 kb)

## Abbreviations

AHRQ: Agency for Healthcare Research and Quality
ANOVA: Analysis of Variance
CCA: Canonical Correlation Analysis
MoH: Palestinian Ministry of Health
PCC: Patient Centered Care
PPRQ: Provider Patient Relationship Questionnaire
RQ: Research Question
SDRS: Socially Desiarable Response Set
