# Supplementary material for: Palestinian doctors’ views on patient-centered care in hospitals
Source: BMC Health Serv Res. 2018 Oct 11;18:766. doi: 10.1186/s12913-018-3573-0 (PMC6180518; doi:10.1186/s12913-018-3573-0)
Supplement: Supplementary file 1 — Palestinian Doctors’ Views on Patient-Centered Care in Hospitals. (DOCX 24 kb) [file 12913_2018_3573_MOESM1_ESM.docx]

# Palestinian Doctors’ Views on Patient-Centered Care in Hospitals

Wasim I. M. Sultan, Mutaz I. M. Sultan, José Crispim

**Doctor’s Survey**

**Dear doctor:**

This survey applies to all doctors working in any Palestinian Hospital. It is a part of a doctorate thesis (Ph.D.) in Business Administration at the University of Minho - Portugal. The study explores the physicians' perspective to implement four aspects of Patient-Centered Care (PCC). On average, answering the questionnaire consumes 7-8 minutes. We do appreciate and acknowledge your cooperation to fill it transparently. The privacy of the collected data is highly respected and applies for academic purposes only.

***Please consider the following in mind:***

1- Submit one response only.

2- Patients included in discussion are neither severe urgent cases nor in chronic disease patients.

3- Pediatricians apply interviews with families.

4- Think about your last interviews with patients during this week.

5- I would appreciate very much if you pass it to your colleagues and promote them to fill it. The questionnaire is available on the given link: <http://bit.ly/22u7XMP>

*Corresponding researcher:* ***Wasim Idris Sultan***

Email: [waseem@ppu.edu](mailto:waseem@ppu.edu)

**Part one:** Demographic and social variables,

*Please circle the statement that fits your job and personal attribute:*

| **● Job Title** | ⃝ Specialist | ⃝ Resident | ⃝ General |
| --- | --- | --- | --- |
| **● Department (Specialty)** | ⃝ Surgery | ⃝ Inter. Med. | ⃝ Obs. & Gyn. |
|  | ⃝ Pediatrics | ⃝ Anesthesia | ⃝ Emergency |
|  | ⃝ Radiology | ⃝ Others | ……………………………………… |
| **● Gender** | ⃝ Male | ⃝ Female |  |
| **● Age** | ⃝ <=35 years | ⃝ > 35 years |  |
| **● Marital Status** | ⃝ Single | ⃝ Married | ⃝ Others |
| **● Hospital Setting where you work** | ⃝ Govern. | ⃝ Non govern. |  |
| **● Years in the hospital.** | ⃝ <= 5 years | ⃝ > 5 years |  |
| **● University of Bachelor Education** | ⃝ Inside Palestine | ⃝ Outside Palestine |  |
| **● Training / communication with patient** | ⃝ Never got training | ⃝ I got training /communication |  |
| **● Patient-Centered Care (PCC)** | ⃝ Never knew about PCC | ⃝ I Know PCC approach. |  |
| **● On average I spend interviewing my patient a time of** | ⃝ 5-10 minutes | ⃝ 10-15 minutes | ⃝ 15-20 minutes |

**Part two:** PCC statements,

Please **tick one circle** for each statement below to show how much you consider it as important: (These 16 questions were adapted from a self-assessment tool developed by Gremigni *et al.* (2016). Scales are modified by the researchers.

|  | **Not at all important** | **Somewhat important** | **important** | **Mostly**  **important** | **Totally important** |
| --- | --- | --- | --- | --- | --- |
| ***● Provide clear information to the patient.*** | ⃝ | ⃝ | ⃝ | ⃝ | ⃝ |
| ***● Turn to the patient in a calm and quiet tone*** | ⃝ | ⃝ | ⃝ | ⃝ | ⃝ |
| ***● Show respect to the patient as a person rather than a case.*** | ⃝ | ⃝ | ⃝ | ⃝ | ⃝ |
| ***● Pay attention to what the patient says.*** | ⃝ | ⃝ | ⃝ | ⃝ | ⃝ |
| ***● Show interest in what the patient feels about his status.*** | ⃝ | ⃝ | ⃝ | ⃝ | ⃝ |
| ***● Show interest in what the patient knows about his disease/prognosis.*** | ⃝ | ⃝ | ⃝ | ⃝ | ⃝ |
| ***● Show interest in what the patient wants from care.*** | ⃝ | ⃝ | ⃝ | ⃝ | ⃝ |
| ***● Show interest in what the patient expects from care in this hospital.*** | ⃝ | ⃝ | ⃝ | ⃝ | ⃝ |
| ***● Understand the emotions that the patient may have.*** | ⃝ | ⃝ | ⃝ | ⃝ | ⃝ |
| ***● Check how illness affects the patient’s daily life activities.*** | ⃝ | ⃝ | ⃝ | ⃝ | ⃝ |
| ***● The doctor acts as putting himself in the “patient’s place”.*** | ⃝ | ⃝ | ⃝ | ⃝ | ⃝ |
| ***● Inspire confidence and security when touching the patient or being nearby.*** | ⃝ | ⃝ | ⃝ | ⃝ | ⃝ |
| ***● Give the patient enough time to ask and talk about his disease.*** | ⃝ | ⃝ | ⃝ | ⃝ | ⃝ |
| ***● Ask the patient questions that allow him to express his point view.*** | ⃝ | ⃝ | ⃝ | ⃝ | ⃝ |
| ***● Give him encouragement and transmit optimism* التفاؤل** | ⃝ | ⃝ | ⃝ | ⃝ | ⃝ |
| ***● Offer the patient opportunity to discuss and share decision making.*** | ⃝ | ⃝ | ⃝ | ⃝ | ⃝ |

**Part three:** Working Environment,

Please **tick one circle** for each statement below to show how much you agree it applies to the hospitals, other doctors, patients, and nurses in your work settings: Items are developed by the researcher.

|  | **Strongly Disagree** | **Disagree** | **Neutral** | **Agree** | **Strongly Agree** |
| --- | --- | --- | --- | --- | --- |
| ***● Hospital culture does not support effective medical interview with patients.*** | ⃝ | ⃝ | ⃝ | ⃝ | ⃝ |
| ***● I am overloaded with work and don’t have enough time for a good interview.*** | ⃝ | ⃝ | ⃝ | ⃝ | ⃝ |
| ***● Other doctors support my good interview and facilitate it.*** | ⃝ | ⃝ | ⃝ | ⃝ | ⃝ |
| ***● Nurses cooperate positively with me to interview patients effectively.*** | ⃝ | ⃝ | ⃝ | ⃝ | ⃝ |
| ***● In general, patients tend to give less information due to social consequences.*** | ⃝ | ⃝ | ⃝ | ⃝ | ⃝ |
| ***● Most patients are not aware of their health status or their disease.*** | ⃝ | ⃝ | ⃝ | ⃝ | ⃝ |
| ***● Few patients judge communication tasks as valued or relevant* ذات علاقة *to healthcare*** | ⃝ | ⃝ | ⃝ | ⃝ | ⃝ |
| ***● I don’t think that communication tasks help in clinical reasoning or improve the quality of medical care.*** | ⃝ | ⃝ | ⃝ | ⃝ | ⃝ |
| ***● I feel satisfied with my work in this hospital.*** | ⃝ | ⃝ | ⃝ | ⃝ | ⃝ |
| ***● I find my job as interesting*** | ⃝ | ⃝ | ⃝ | ⃝ | ⃝ |
| ***● I prefer to be formal rather than warm and friendly.*** | ⃝ | ⃝ | ⃝ | ⃝ | ⃝ |

**Part four:** Personal traits,

|  | **Definitely False** | **Mostly False** | **Neutral** | **Mostly True** | **Definitely True** |
| --- | --- | --- | --- | --- | --- |
| ***● I am always courteous مهذب even to disagreeable* مكروهين *people.*** | ⃝ | ⃝ | ⃝ | ⃝ | ⃝ |
| ***● There have been occasions when I took the advantage* استغلّيت *of someone.*** | ⃝ | ⃝ | ⃝ | ⃝ | ⃝ |
| ***● I sometimes get resentful* ممتعض *when I don’t get my way.*** | ⃝ | ⃝ | ⃝ | ⃝ | ⃝ |
| ***● No matter who I am talking to, I am always a good listener.*** | ⃝ | ⃝ | ⃝ | ⃝ | ⃝ |
